# 60 years of Charnley–Muller Alivium hip prosthesis: the revision percentage and tribo-corrosion sequelae after a mean of 27 years

**DOI:** 10.1007/s00402-023-04824-y

**Published:** 2023-03-16

**Authors:** Ingrid Milošev, Rihard Trebše, Andrej Cör, Vesna Levašič

**Affiliations:** 1 Research Department, Valdoltra Orthopaedic Hospital, Jadranska C. 31, 6280 Ankaran, Slovenia; 2 Department of Physical and Organic Chemistry, Jožef Stefan Institute, Jamova c. 39, 1000 Ljubljana, Slovenia; 3 Faculty of Medicine, University of Ljubljana, Korytkova 2, 1000 Ljubljana, Slovenia; 4 University of Primorska, Titov Trg 4, 6000 Koper, Slovenia

**Keywords:** Charnley–Muller hip prosthesis, Wear, Wear particles, Revision percentage

## Abstract

**Introduction:**

The main aim was to analyse the series of 29 collected cemented Charnley–Muller Alivium retrievals with the meantime in situ of 27 years. In addition, the revision rate of 1425 Alivium prostheses implanted at our institution between 1977 and 1992 was calculated.

**Materials and methods:**

The revision percentage of the Alivium cohort was calculated up to 45 years of follow-up and compared to that of all total hip arthroplasties (THAs) implanted in the same period (No. 5535). Metal and polyethylene retrieved components were inspected in 29 cases for wear damage and roughness. Wear particles were retrieved from periprosthetic tissue using digestion protocols and their composition, morphology, and size distribution were investigated. Periprosthetic tissue was analysed histologically.

**Results:**

The revision percentage of the Alivium cohort was 16% at 45 years of follow-up. It was comparable to all the THAs implanted at the same time (18%). The shape of polyethylene particles isolated from periprosthetic tissue corresponded to the wear pattern on polyethylene cups. Polyethylene particles were the main wear product, with the majority (68%) of particles smaller than 0.1 µm. Metal particles were rare with two types: CoCr and Cr based. Histological analysis showed that in 14 out of 18 specimens, the metal particles were graded + 1, reflecting that the metal loading in the periprosthetic tissue was low.

**Conclusions:**

Our study represents valuable data not reported previously on the survival rate of Charnley–Muller prostheses at 45 years of follow-up and a unique insight into the collected retrievals from the materials’ point of view.

## Introduction

November 2022 will mark the 60th anniversary of the first implantation performed by Prof. Sir John Charnley [1]. Throughout the last 6 decades of use, cemented prostheses evolved through various designs, materials, collar types, surface finishes, and surgical techniques [2]. In the early era of total hip arthroplasty, only a limited choice of implant types was available. The clinical outcomes of Charnley THAs implanted in the 1970s and 1980s are well documented [3]. Charnley–Muller THA, launched in 1966, was a continental version of the classic Charnley THA; the related long-term results are rare [4]. Lessons learned from the oldest implants used (e.g. Charnley-type, Lubinus, Stanmore, and Exeter) have been of paramount importance for the development of contemporary THAs. However, there is a lack of data regarding the impact of wear and corrosion products of the oldest implant, in contrast to novel types of THAs [5–7]. In the 1960–1980s, retrievals were either not collected, or the availability of materials characterisation had not been fully established yet.

The main aim of this study was to analyse the large series of collected cemented Charnley–Muller Alivium retrievals with the meantime in situ of 27 years. In addition, we looked at the whole cohort of patients implanted between 1977 and 1992 at our hospital and determined the percentage of all revisions. The main hypotheses were: (1) the surfaces of cemented Alivium CoCrMo THAs are subject to severe corrosion and morphological changes due to the long implantation period, and (2) the revision percentage of Alivium THAs over 40 years is comparable to the group including all THAs in the same period.

## Patients and methods

### Implant

**Fig. 1:**
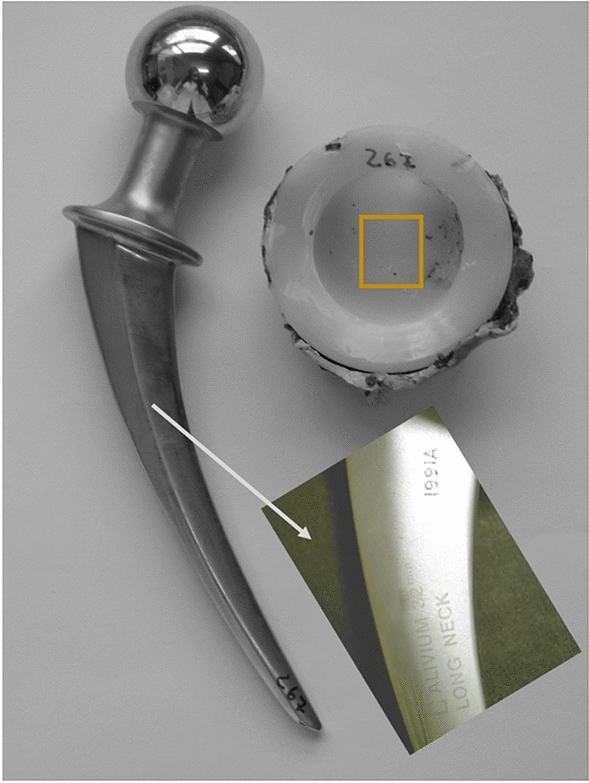
Charnley–Muller Alivium monoblock hip implant with high-density polyethylene cup. Both femoral and acetabular parts are cemented. Inset shows the Alivium trademark

Alivium is a vacuum melt, vacuum cast total joint prosthesis (Zimmer Orthopeadics Ltd, Great Britain) made of cobalt–chromium–molybdenum alloy (complies with British Standard (B.S): 3531: 1968 and American Society for Testing and Materials (ASTM): F-75: 1967). It has been used in combination with high-density (H.D.) polyethylene acetabular cups since 1966. The diameter of the femoral head was 32 mm (Fig. 1). As indicated by the manufacturer, the implant was designed to minimise the risk of dislocation with minimal influence on the wear rate of the acetabular component.

### Operative technique

The operative procedures were performed through a direct lateral [8], anterolateral [9], or posterolateral surgical approach [10]. Perioperative administration of first-generation cephalosporins was implemented in our institution in 1986, while heparin was introduced at the end of 1983, so the vast majority of the patients from the cohort have not received these prophylactic regimes.

### Patient cohort

We analysed the cohort of patients with THAs from January 1, 1977, to December 31, 1992, based on operation protocol books. Therefore, this study is retrospective. There were 5,535 THAs of different producers. Among those, 1,425 were Alivium Zimmer THAs. To calculate the revision percentage, we have linked the whole cohort to revision operation data retrieved from operation protocol books (till 2002), and Valdoltra Arthroplasty Registry (from 2002 on). Revision percentage was calculated as the number of revision operations in the targeted group relative to the number of primary operations in the targeted group. The limitation of this procedure is that we could not provide the data on these patients’ deaths, so it was not possible to make the Kaplan–Meier survival curve. However, it was almost impossible to retrieve personal data for such a large cohort from 45 years ago. The revision percentages of the Alivium cohort group and the group with all THAs were compared using a t-test (Excel MS for Windows). Revision percentages were calculated at 10, 20, 30, 40, and 45 years of follow-up. Although we could not retrieve data on the patient’s death, it is assumed that later than 20-year follow-up, the number of dead patients exceeds the number of failures, thus the statistical uncertainty of the numbers would become larger.

The number of patients lost to follow-up was probably less than 5%. Namely, according to the National Arthroplasty Registry of Slovenia [11], only 6% of revisions of Valdoltra’s patients are carried out in other hospitals. In the past, this number was even smaller.

### Retrievals’ analysis

In the period from September 2002 to September 2015, we collected 29 Alivium monoblock implants retrieved from 29 patients. The primary operations for these patients dated from November 1977 to February 1992. Data on the patients, including age, gender, initial diagnosis, details regarding the implant, the reason, and the date of revision, were retrospectively retrieved from the hospital registry [11]. The analysis of the clinical and radiographic data from all the patients in the database was beyond the scope of the present study.

21 patients were female (72%), and 8 were male (28%). 16 hips were implanted on the left side and 13 on the right side. The diagnosis at the primary operation was osteoarthrosis.

### Analysis of retrieved implants

#### Roughness and surface analysis

The roughness of the retrieved femoral heads was measured at 4 sites on the surface with a diamond stylus profilometer Talysurf Hobson Form (Talysurf Series 2; Leicester, UK) with the use of a 2-mm evaluation length and a 0.25-mm cutoff length. The measurement spots were at the dome, at 45° and 90° from the dome, and representative sites within the worn zone. At each zone, measurements were carried out at four sites sized 1 mm^2^. The results were expressed as the average surface roughness (*R*_a_) and the maximum peak-to-valley height (*R*_t_) for each zone. At selected sites, the surface was examined with a confocal microscope (Zeiss Axio CSM 700) and field emission scanning electron microscope SEM (JEOL JSM 7600F, Tokyo, Japan) at a beam acceleration voltage of 15 kV in low secondary electron image (LEI) mode. Before analysis, the samples were coated with a thin carbon layer to reduce charging.

#### Isolation and analysis of wear particles from periprosthetic tissue

The retrieved periprosthetic tissue samples obtained during the revision surgery were frozen and then later used to isolate wear particles. The protocol used for the polyethylene wear particle isolation was a modified version of Campbell et al. [5]. Tissue samples were shaken overnight on the shaker in a mixture of chloroform and methanol (2:1) to extract lipids. Then, the tissues were digested in 5 M sodium hydroxide for four hours at 65 °C. The digest was then centrifuged at 6000 rpm (revolution per minute, the number of full rotations completed in 1 min) for 1 h over a sucrose gradient (5% sucrose). The dense upper layer that contained polyethylene particles was collected and hydrolysed for 1 h at 80 °C and then centrifuged with isopropanol (1 h at 6000 rpm). Polyethylene particles formed a thin white band on the top of the isopropanol solution; this layer was transferred to a clean vial. The sediment at the bottom of the tube contained heavier particles, i.e. metal, organic, and cement particles. Sediment was collected, carefully rinsed, and transferred to a clean vial. Collected samples were stored in a cool place.

Isolation for metal particles was based on the ASTM F561-05 [12]. Briefly, tissue was digested in 1 mL distilled water and 150 µL of 2.5% sodium dodecyl sulphate (SDS), boiled for 12 min, centrifuged at 13,400 rpm, and rinsed three times with phosphate buffer. After ultrasonication, the supernatant was mixed with papain solution (1 mL phosphate buffer, 100 µL pure papain, 3.26 mg N-acetylcysteine, 9 ml ultrapure water) and incubated 24 h at 65 °C. After centrifugation at 13,400 rpm, the supernatant was centrifuged at 13,400 rpm, resuspended in 1 mL of 2.5% SDS, and boiled for 10 min. After washing with 1 mL of 50 mM Tris–HCl (pH = 7.6), the sample was incubated with 400 µg proteinase K in 1 mL TRIS–HCL for 24 h at 55 °C. Afterwards, the particles were re-centrifugated, resuspended in 1 mL SDS, boiled for 10 min, and washed with Tris–HCl and distilled water. Particles were stored in 100% ethanol at 4 °C.

## Microscopic analysis

Before the microscopic analysis, vials were ultrasonicated for 5 min; a small volume of isolated polyethylene particles was filtered through membrane nucleopore polycarbonate filter papers (Costar, Pleasanton, California) with a pore size of 0.2 µm. Filter papers with polyethylene particles were sputter-coated with gold or graphite and analysed with a scanning electron microscope SEM (JEOL JSM 5800, Tokyo, Japan) at beam accelerating voltage of 15 kV in secondary electron (SE) imaging mode. For each sample, three to five images on different fields were photographed at different magnifications, allowing the differentiation of particles.

### Size distribution of polyethylene particles

To perform the analysis of the size distribution of isolated polyethylene particles, the representative SEM images of polyethylene particles taken at the magnification of ×5000, ×7000, or ×10,000 were analysed. First, each particle on the SEM image was pointed manually. This step was time-consuming but was required due to a strong agglomeration of the particles. Once all the particles were manually recognised, the image was scanned and then analysed using the image processing and analysis software (Image-Pro PLUS; Media Cybernetics, Bethesda, USA). The particle size was defined as the maximum diameter. Particle size was defined by the equivalent circle diameter (ECD): the diameter of a circle with an area equivalent to the area of the particle. It has units of length. The percentage of particles in each size range was calculated.

### Histological analysis

Interface membranes from the femoral stem and acetabular cup were taken during the revision arthroplasty, fixed in 10% formalin, and embedded in paraffin. From paraffin blocks, 5 µm-thick serial sections were prepared and stained with hematoxylin and eosin, Perls, and Giemsa histochemical staining methods and analysed with a light and polarising microscope (Eclipse 80i; Nikon, Tokyo, Japan) equipped with a digital camera (DXM1200-F; Nikon). Particle categories were scored according to size, shape, colour, and birefringence in polarised light [13]. Diffuse and perivascular lymphocytic infiltrates were also evaluated and scored.

## Results

### Data on the whole cohort and revision percentage

**Fig. 2:**
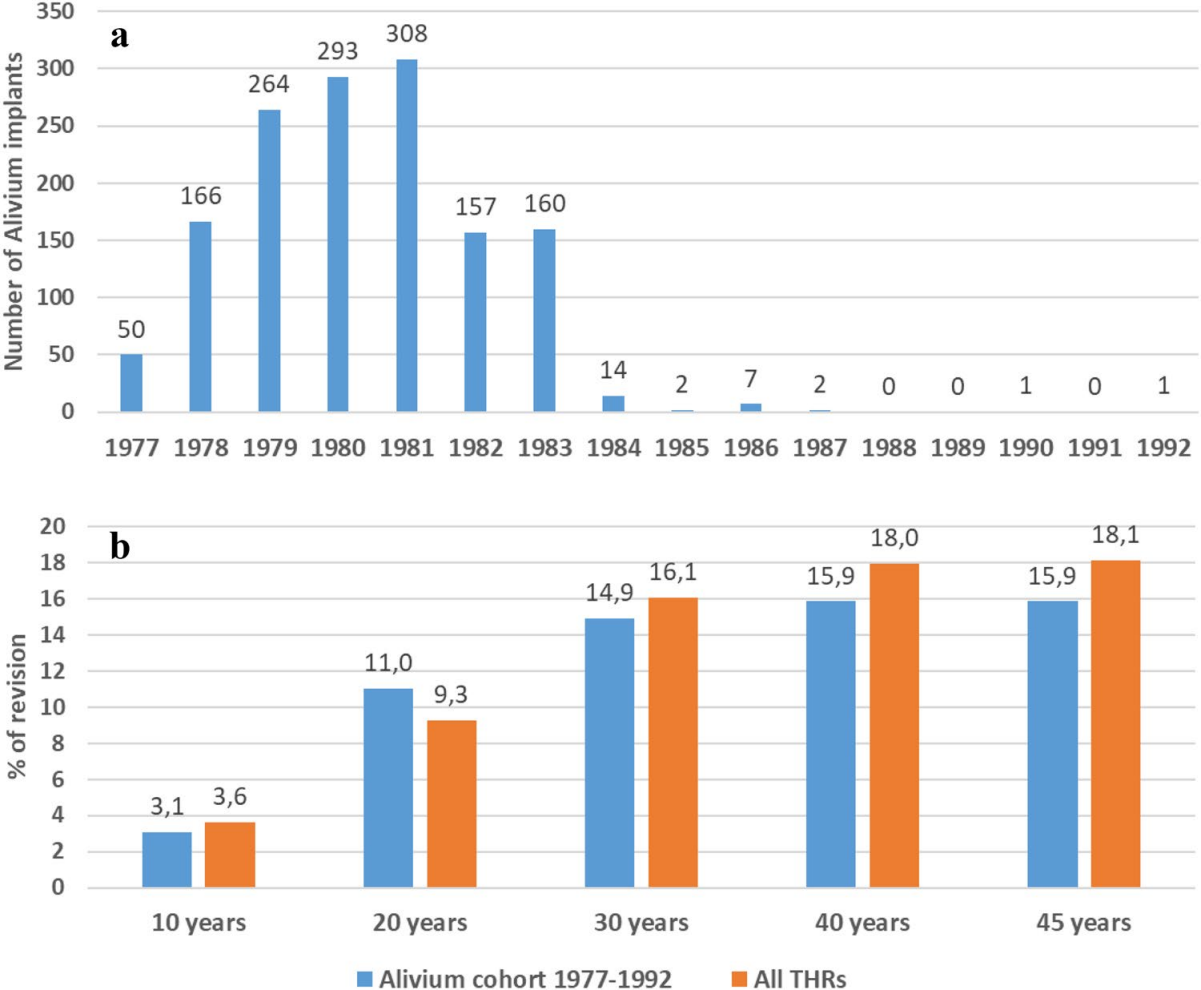
**a** Number of Alivium THPs implanted at Valdoltra Orthopaedic Hospital from 1977 to 1992. **b** Revision percentage at 10, 20, 30, 40 and 45 years for the Alivium cohort compared with the revision percentage for all THAs implanted at Valdoltra Orthopaedic Hospital in the same period

The number of Alivium THAs implanted from 1977 to 1992 is shown in Fig. 2a. Initially starting with 50 THAs in 1977, the number peaked in 1981 when 1081 THAs have been implanted. Up to the year 1983, the number reached 1,398. Between 1984 and 1992, only an additional 27 THAs were implanted, making the total cohort of 1,425 Alivium THAs.

The revision percentage calculated for the Alivium cohort at 10-, 20-, 30-, 40- and 45-year follow-up is presented in Fig. 2b. It ranged from 3.1% (44 revised THAs) at 10 years, to 11% (157 THAs), 14.9% (213 THAs), 15.9% (226 THAs) and 15.9% (226 THAs) at 20, 30, 40 and 45 years, respectively. The same data are presented for all the THAs implanted in the same period at our hospital. Similar values were obtained, ranging from 3.6% (122 revised THAs) at 10 years, to 9.3% (512 THAs), 16.1% (891 THAs), 18% (995 THAs), and 18.1% (1004 THAs) at 20, 30, 40 and 45 years, respectively. No statistically significant difference was obtained between the two groups (*p* = 0.30).

### Patients’ demographic data and data on revision operations in the collected retrieved implants

For 29 retrieved Alivium THAs, the median patient age at the time of primary operation was 51 years (range 34–80) and at the time of revision operation, 80 years (range 64–95). 14 patients had hip implants on both sides.

The reasons for the revision operation of stems were as follows: loosening of the femoral component (2), loosening of the acetabular component (2), loosening of both components (15), periprosthetic fracture (7), fracture of the implant (1), and septic loosening (2). In the cases of fractures of the femur, the implant was already loosened on the femoral or both sides. The average time in situ for the Alivium stems was 27 years (range 11–34, SD 6).

At the revision operation of the stem, 19 out of 29 patients had the original acetabular component implanted at the primary operation. 15 out of 19 were revised during the revision operation. The average time in situ for 15 original cups was 25 years (range 16–30, SD 4). The remaining 4 cups were not revised at the revision operation of the stem and were left in situ.

The remaining 10 out of 29 patients underwent revision of the acetabular component before the revision of the femoral component. The reason for the revision was loosening at a mean of 15 years (range 9–21, SD 4). Out of these 10 patients with revised acetabular cups, 4 cups were revised at the revision operation of the stem, and 6 were left in situ.

**Fig. 3:**
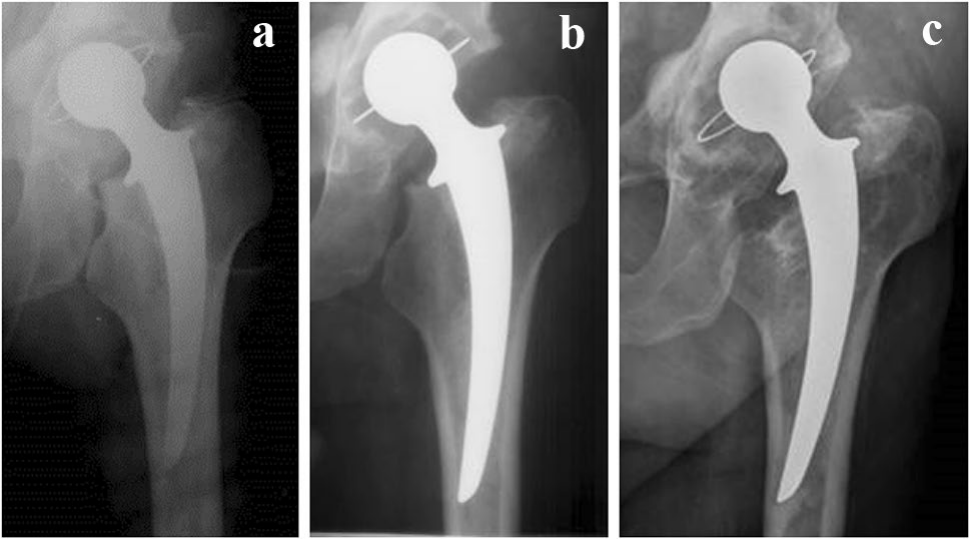
X-ray image of Alivium Charnley–Muller prosthesis after the primary operation (**a**), 3 months post-op (**b**), and 31 years post-op, preceding the revision (**c**)

Figure 3 presents representative X-ray images of the patient with the longest time in situ period with Alivium THA. After implantation in March 1982 and 3 months post-op, there was an optimal valgus position of the stem and a good position of the socket with a homogeneous, uninterrupted cement mantle around both components (Fig. 3a, b). After 31 years, eccentric acetabular wear, osteolytic lesions around both components, and loosening of the stem with slight subsidence are noted (Fig. 3c). After 33 years, the implant was revised with a cementless, press-fit socket and a revision rectangular tapered stem. The fenestration needed for removing the cement distal to the tip of the primary implant was fixed with cerclage tapes.

### Analysis of retrieved implant components

#### Wear damage on retrieved components

**Fig. 4:**
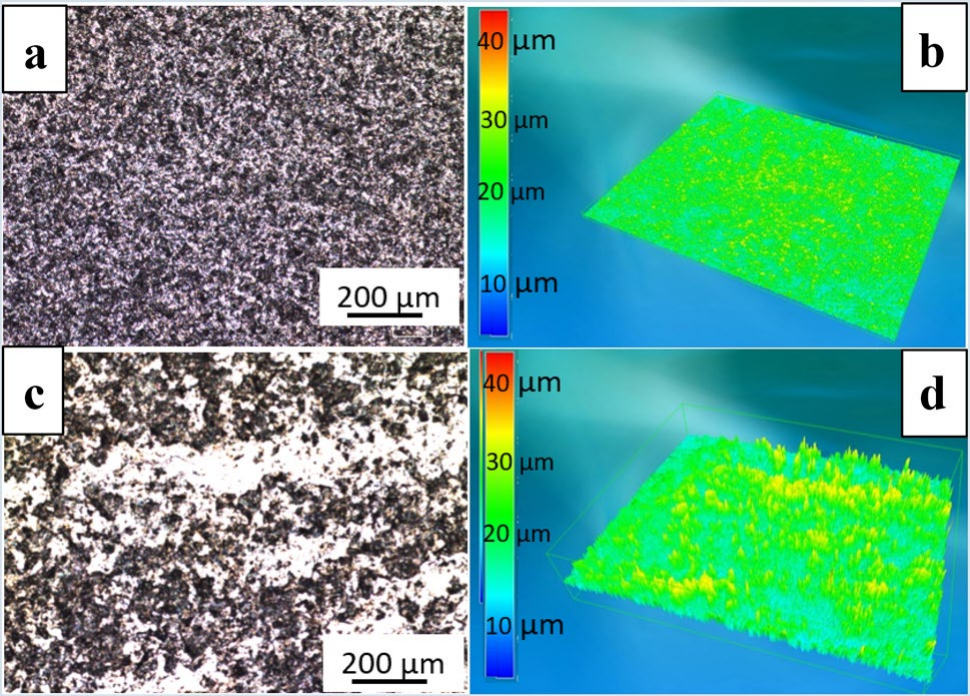
Typical **a**, **c** 2D and **b**, **d** 3D images of unworn and worn areas on retrieved Alivium femoral heads. The difference in surface roughness is evident in 2D images; in 3D images, the colour scale defines the magnitude of roughness

**Fig. 5:**
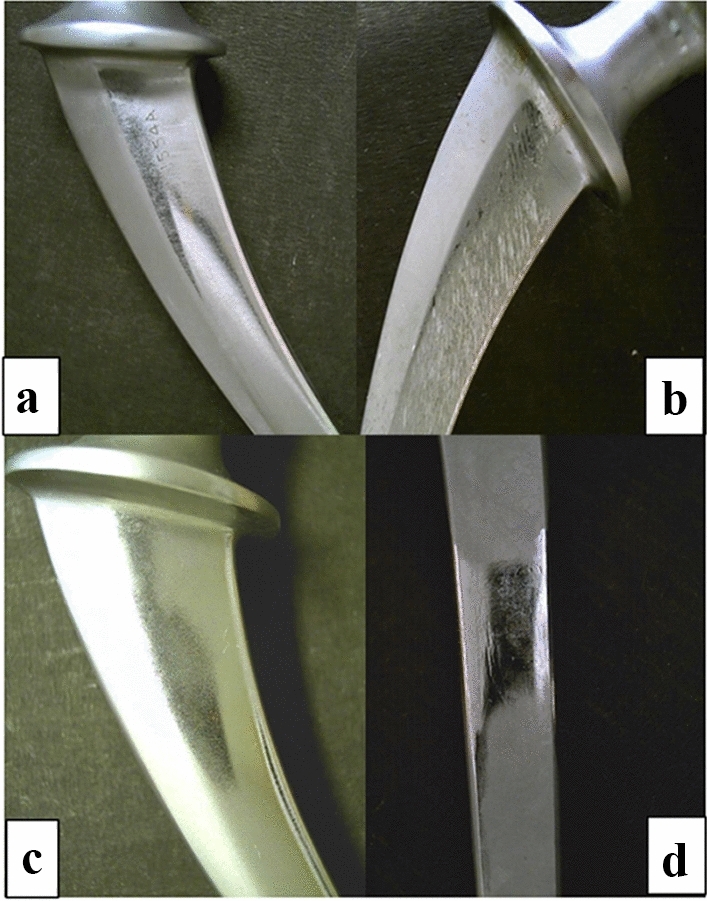
Examples of **a** wear marks, **b** scratches, and **c**, **d** polishing marks on retrieved Alivium femoral stems

**Fig. 6:**
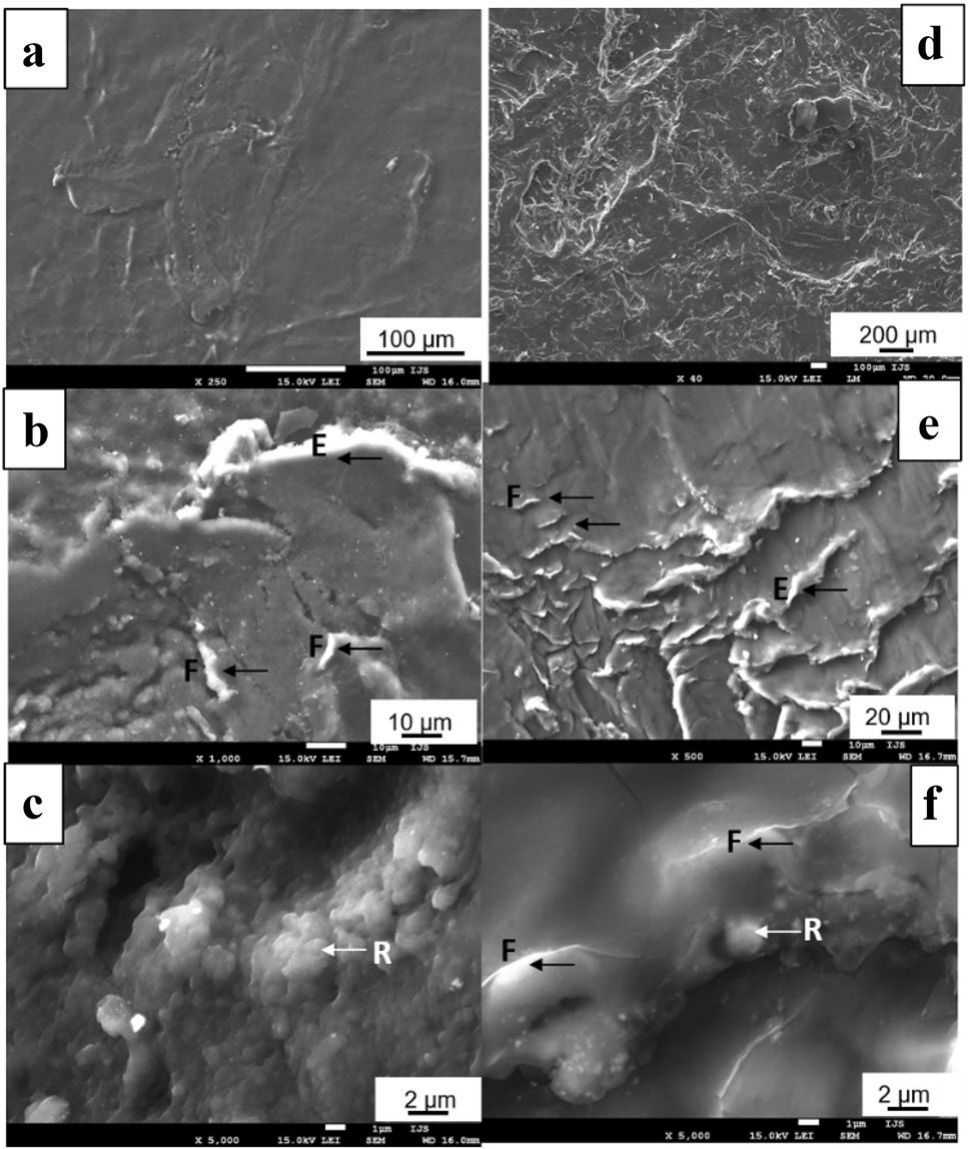
SEM images of the interior of retrieved high-density polyethylene cups implanted with Alivium femoral stems. Examples of two cups are presented in each vertical column, starting from low to high magnification. Typical wear marks are noted by arrows: micrometre-sized fibrils (*F*), elongated, several tens of micrometres (*E*), and round particles (*R*)

Roughness was measured on 17 retrieved femoral heads. Surface roughness was more than doubled in the worn area compared to the dome or non-worn area (Table 1). 2D and 3D images of sites in non-worn and worn sites are presented in Fig. 4. Typical wear marks, scratching, and polishing of the femoral stems are presented in Fig. 5. Wear damage on retrieved polyethylene components exhibit different shapes: fibrils and larger, elongated micrometre-sized particles, and nanometre-sized round particles (Fig. 6).

**Table 1:** The roughness of 17 retrieved femoral heads measured at different zones given as mean ± standard deviation (min to max value). *R*_a_ is the average surface roughness and *R*_t_ is the maximum peak-to-valley height

Roughness	Dome	45°	90°	Wear
*R*_a_/µm	0.066 ± 0.056 (0.020–0.236)	0.050 ± 0.048 (0.018–0.212)	0.057 ± 0.035 (0.023–0.138)	0.142 ± 0.102 (0.024–0.405)
*R*_t_/µm	0.759 ± 0.460 (0.168–1.426)	0.584 ± 0.490 (0.160–2.070)	0.610 ± 0.440 (0.217–1.889)	1.470 ± 1.413 (0.252–6.138)

#### Analysis of wear particles

**Fig. 7:**
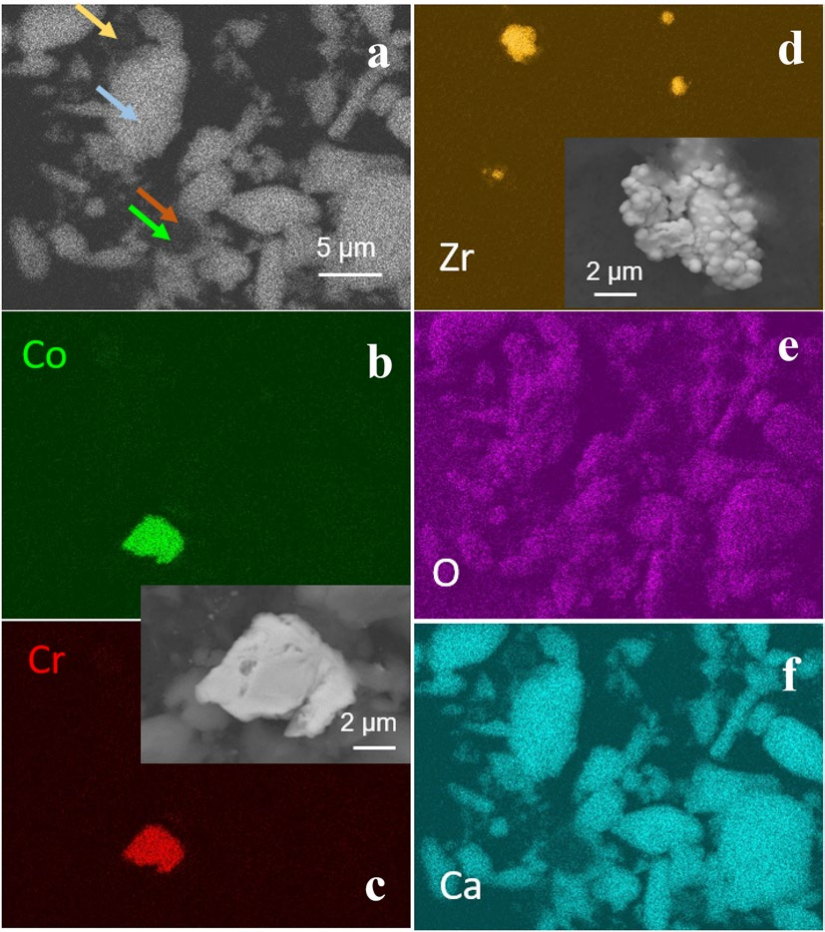
Morphology and composition of wear particles isolated from periprosthetic tissue by alkaline protocol. **a** SEM BSE image showing particles differing in composition (heavy elements appear darker than bright ones), as pointed out by arrows. **B**–**f** EDS compositional maps of the SEM image in Co, Cr, Zr, O, and Ca. Arrows of the same colour note the presence of particles in the SEM image in (**a**). SEM BSE image of the metal particle is shown in the inset in (**c**). SEM BSE image of ZrO_2_ particles added to polymethylmethacrylate bone cement is shown in the inset in (**d**)

**Fig. 8:**
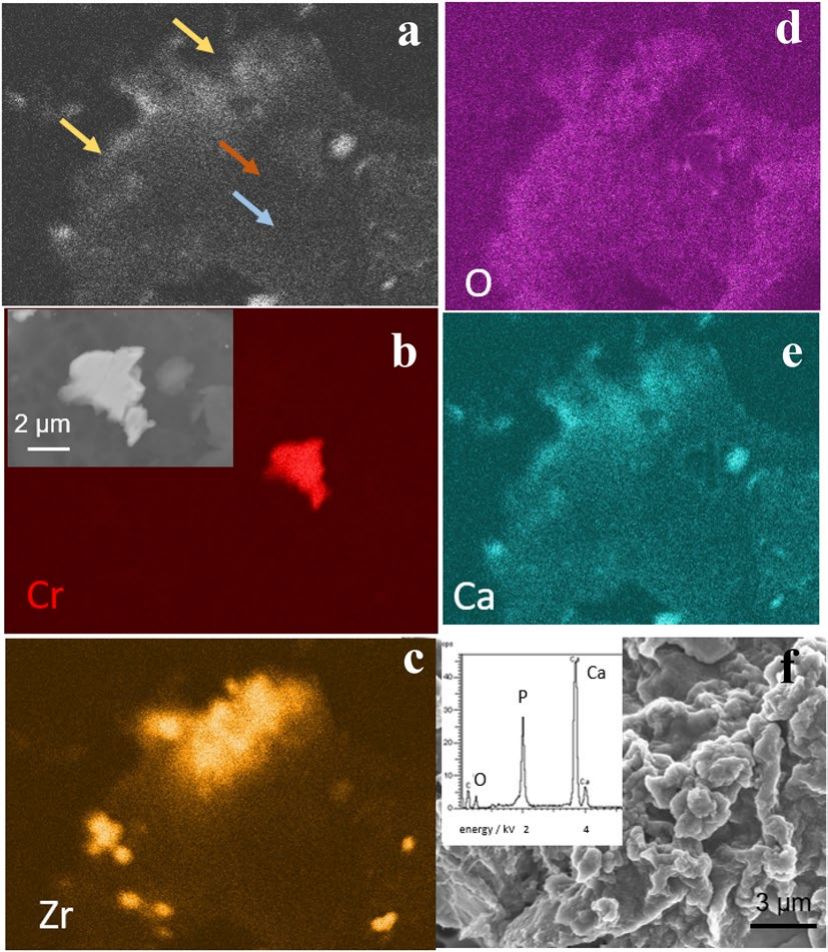
Morphology and composition of wear particles isolated from periprosthetic tissue by alkaline protocol. **a** SEM BSE image showing particles differing in composition (heavy elements appear darker than bright ones), as pointed out by arrows. **b**–**f** EDS compositional maps of the SEM image in Co, Zr, O, and Ca. Arrows of the same colour note the presence of particles in the SEM image in (**a**). SEM BSE image of the metal particle is shown in the inset in (**b**). **f** SEM SE image and EDS spectrum of calcium phosphate particles

Metal particles were only rarely isolated from the tissue. When using the alkaline protocol, no metal particles were detected. Metal particles were detected in one sample only when using the enzymatic digestion protocol. Different types of particles are presented in Figs. 7a and 8a. In BSE SEM images, heavier particles appear brighter than lighter particles. EDS compositional maps resolve particles on the SEM image according to their composition. Two types of metal particles were identified: CoCr-particle (Fig. 7b, c) and Cr-particle (Fig. 8b). Particles are plate-like shaped and relatively large.

ZrO_2_ particles also appear bright on the SEM images (Figs. 7d, 8c). These particles, added to polymethylmethacrylate bone cement to assure radio-opacity, have globular morphology (inset in Fig. 7d). The most abundant and very bright particles are calcium orthophosphate (Figs. 7f, 8e). The ratio Ca/P, determined from the intensity of EDS signals, is 1.67 and follows Ca-phosphate. Oxygen is present throughout the image since it is a consisting element of all the particles (Figs. 7e, 8d).

**Fig. 9:**
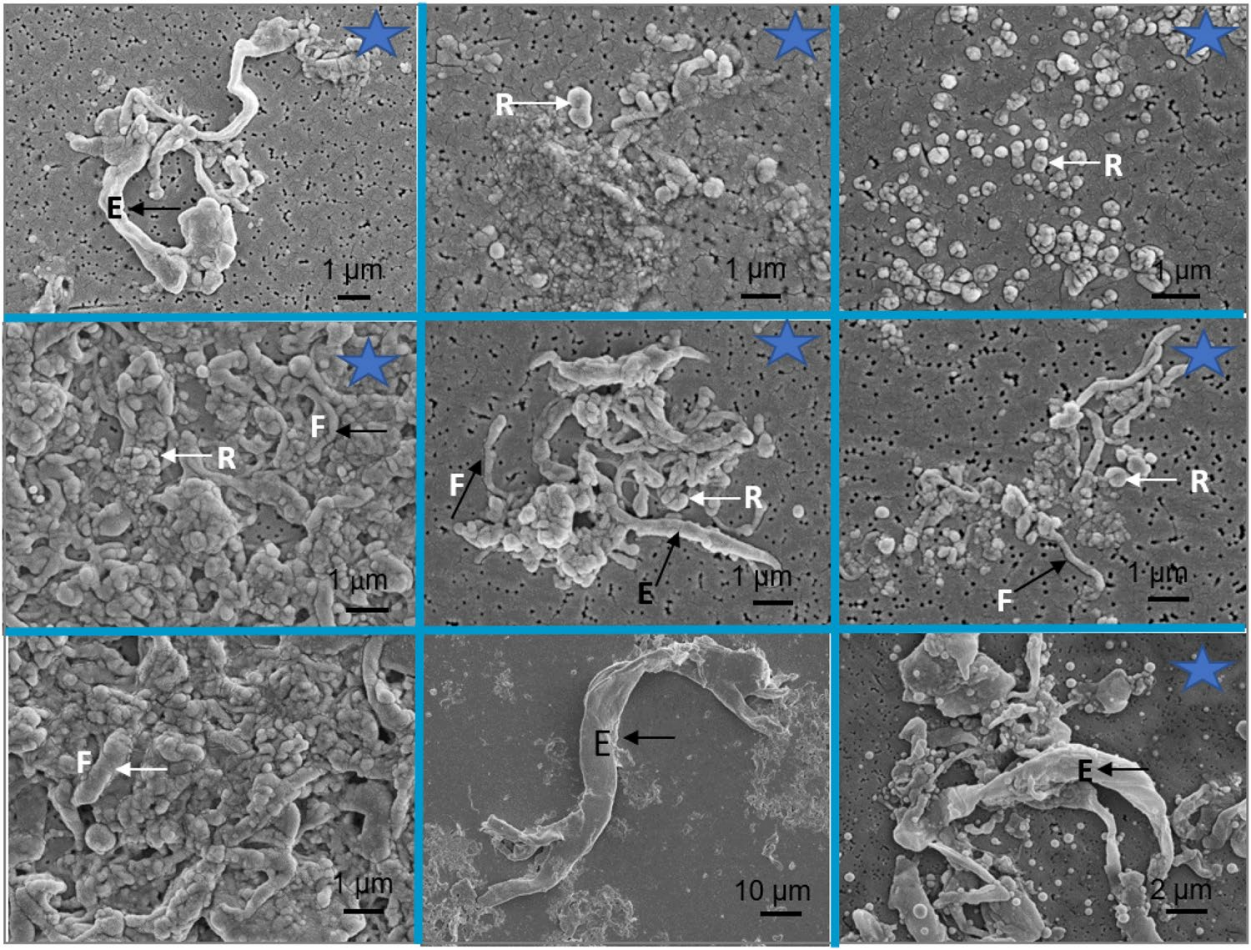
SEM images of polyethylene particles isolated from periprosthetic tissue of cemented high-density polyethylene cups implanted with Alivium stems. Particles were isolated using an enzymatic digestion protocol. The typical shapes of particles are noted by arrows: micrometre-sized fibrils (*F*), elongated, several tens of micrometres (*E*), and round particles (*R*). The shape of isolated particles can be related to the shape of wear marks on retrieved polyethylene cups (Fig. 6). Stars denote the images for which the size distribution of particles was analysed (Fig. 10)

Polyethylene particles were successfully isolated from tissue samples of 12 patients and analysed by SEM (Fig. 9). The majority of particles were oval to round in shape (*R*), thin fibrils (*F*), and large, several micrometres long elongated particles (*E*). The shape and size of isolated particles are following the morphology of the features observed within the interior of retrieved cups (Fig. 6): black arrows denote elongated and fibril particles, and white arrows round particles.

**Fig. 10:**
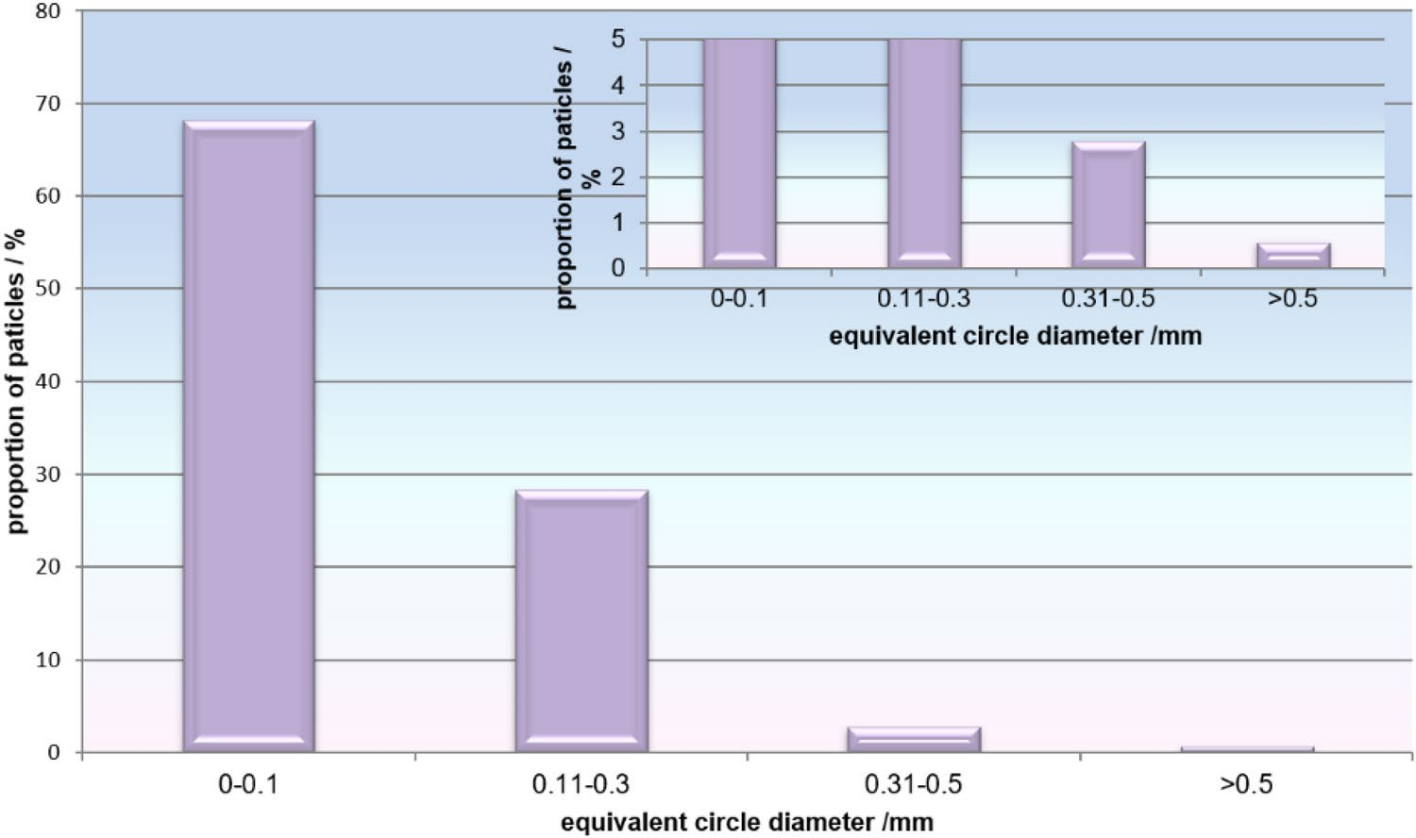
Size distribution of polyethylene particles isolated from periprosthetic tissue of cemented high-density polyethylene cup implanted with Alivium stems. Inset shows an enlarged y-scale

The size distribution of isolated polyethylene particles (Fig. 10) shows that the majority (68%) of particles were smaller than 0.10 µm; 28% of particles were sized between 0.11 and 0.3 µm. Therefore, almost 97% of particles (2034 out of 2106) were sized ≤ 0.30 µm (Fig. 10). Only about 3% of particles (59 out of 2106) were between 0.31 and 0.5 µm and only 0.6% (13 out of 2106) were larger than 0.5 μm.

#### Histological analysis

**Fig. 11:**
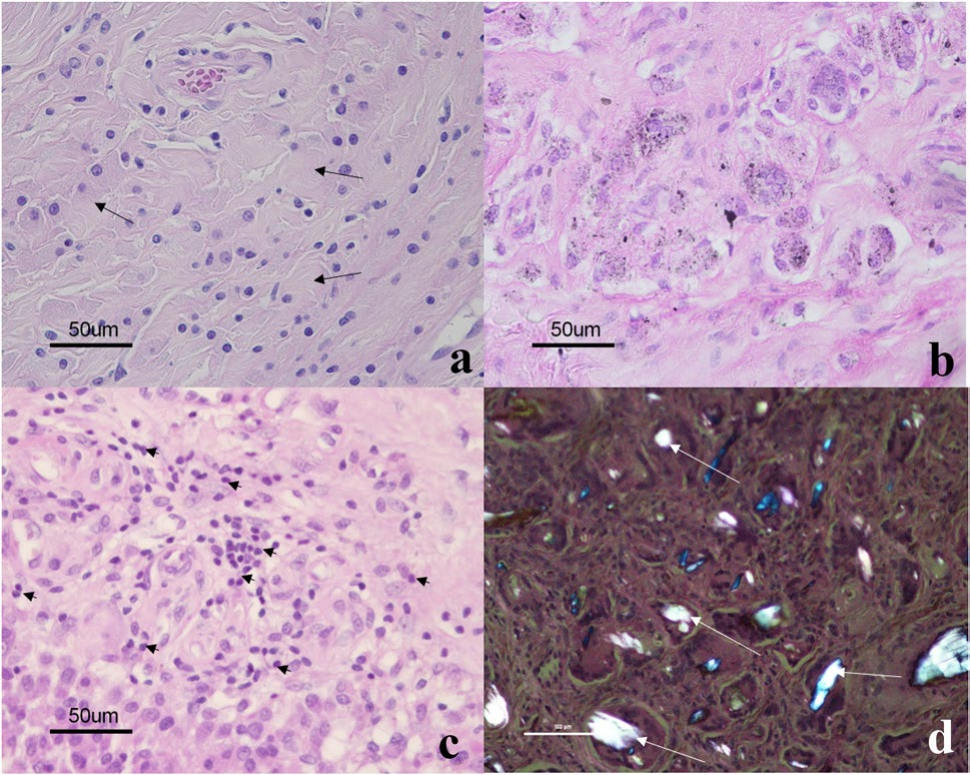
Histological pictures of periprosthetic tissue. **A** slate blue cytoplasm of macrophages (arrows) reflects metal debris below the resolving power of the light microscope; **B** macrophages with more than 10, clearly visible black granules: **C** diffusely distributed lymphocytes (small arrows) between macrophages; **D** polyethylene wear particles (white arrows) as seen with polarised light microscopy (hematoxylin and eosin staining)

Histological samples were collected from 18 of the 29 revised hips. Only aseptic loosening cases were analysed histologically [13]. In these 18 specimens, we observed granulomatous tissue with macrophages with mild metal particles, i.e. only 4 specimens graded + 2 (dusty black macrophages with some visible black granules) (Fig. 11b), whereas most specimens, 14 (78%), were graded + 1 (greyish blue macrophages, because metal particles in the cytoplasm of macrophages were below the resolving power of the light microscope) (Fig. 11a). None of the specimens graded + 3 (jet black histiocytes).

In 8 (44%) out of 18 specimens, giant cells with large polyethylene particles observed under the polarised microscope were found (Fig. 11d). In the rest 10 specimens, only small polyethylene particles were observed under polarised light microscopy ingested by macrophages (starry sky phenomena). Necrosis was observed in all analysed specimens: 9 graded + 1 (1–2 mm of necrosis or necrobiosis/slide), 3 graded + 2 (3–9 mm of necrosis or necrobiosis/slide), and 6 graded + 3 (> 1 cm of necrosis or necrobiosis/slide).

Lymphocytes as chronic inflammatory cells were rarely noticed. Only 2 out of 18 specimens (11%) graded + 2 (10–49 lymphocytes/HPF) (Fig. 11c); 5 (28%) were graded + 1 (1–9 lymphocytes/HPF) and the remaining 11 (61%) specimens graded 0 for lymphocytes. Only diffusely distributed lymphocytes with no large perivascular aggregates and lymphoid follicles were seen.

## Discussion

In this study, we applied retrieval analysis together with the hospital arthroplasty register [11] to attain integrated information on the failure mechanisms and revision rate of a large series of Alivium THAs. This approach has been proven to improve the surveillance of the THA’s performance [14–16]. We collected 29 retrieved Alivium hip prostheses and tissue samples with a mean time in situ of 27 years (range 11–34). Through the collected samples, this study offers a unique opportunity to analyse the implant status and tissue features after 35 years of function. The first hypothesis of this study was that the surface of cemented Alivium CoCrMo THAs is subject to severe corrosion and morphological changes due to the long implantation period. However, based on the analysis of the collected retrievals, this hypothesis can only be partially confirmed. Polyethylene particles were the main wear product, with the majority (68%) of particles smaller than 0.10 µm. The analysis of the interior of a revised UHMWPE liner revealed the origin of different shapes and sizes of particles, including round, fibrils, and elongated, which are related to different mechanisms [17] of the wear process (Fig. 6). The shape of isolated particles can be well correlated with the pattern of wear damage on retrieved polyethylene cups: longitudinal cracks lead to the formation of fibrils and larger, elongated micrometre-sized particles, while nanometre-sized round particles are mainly formed at the sites free of cracks and sites exposed to higher polishing [18]*.* For the high-density polyethylene cups used in this study, the type of sterilisation was not specified. However, the majority of cups produced in the 1970s were gamma-sterilised. This procedure caused higher wear against the femoral metal head compared to later versions of polyethylene [19, 20]. In addition to polyethylene particles, metal, and cement particles were generated due to the relative motion of bearing surfaces against each other and metal femoral stems against the cement mantle, respectively. Cement also contains ZrO_2_ particles added for radio-opacity. Small metal particles were not found; only large metal particles were isolated. Metal wear particles are known to be much smaller than polyethylene particles [12, 21] and, therefore, difficult to isolate. Although we followed the enzymatic protocol recommended for the isolation of metal particles which is less damaging than, e.g. alkaline protocol [21], no small, nanometric-size metal particles were isolated. Histological analysis shows that in 14 out of 18 cases, metal particles were graded + 1, indicating that the metal loading was low. Only micrometric-size metal particles of two types were detected. A possible explanation for the existence of two types of particles, CoCr and Co based, is that initially, the CoCr-particles are removed by wear. Once released, Co may be preferentially dissolved due to its lesser corrosion resistance compared to Cr [22] leaving Cr-remnant particles. Post-mortem analysis of eight Müller well-functioning straight stems after a mean of 9.6-year follow-up revealed a large amount of polyethylene particles, as well as metal and zirconia particles derived from the stem and cement mantle, respectively [23].

The second hypothesis of this study was that the percentage of revised Alivium THAs at 45 years is comparable to the group including all THAs. Twenty-nine collected Alivium THAs represent 13% of all revised Alivium prostheses in 45 years (total No. 226). The comparison of the revision percentages of the Alivium cohort and all implants operated in the same period shows no statistically significant difference, i.e. the revision percentage at 45-year follow-up was 15.9% and 18.1%, respectively. The second hypothesis is, therefore, confirmed.

For the sake of comparison with literature data, we calculated the percentage of prostheses still in situ as the difference between 100% and the revision percentage. Due to the limitation of not including patients’ deaths in the analysis, the real survival curve and survival rate could not be determined. We referred to these values as survival percentages; therefore, the survival percentages after 45 years were 84.1% and 81.9%, respectively.

Long-term clinical outcomes of cemented implants have been published by national registries and different prospective and retrospective studies with follow-up ranging from 5- up to 35-year. Charnley THAs, implanted between 1970 and 1972, using a trochanteric osteotomy approach, achieved a survival rate of 78% at 35 years as revision for any reason as the endpoint [3]. In another study, the Charnley THAs, implanted between 1972 and 1976 and operated in the posterior approach, achieved 93% survivorship and 10 years and 73% at 30 years [24]. A prospective, single-surgeon series with 25 years of follow-up was published aiming for the difference in cementing technique [25]. No statistically significant difference was reported between the two groups, with a survival rate of 80% and 81% years at 25 years. In these studies, the average patient age was 60 [24], 65 [3], and 69 years [25]. The results published for patients under 50 years also have satisfactory long-term function and durability: 63% of the original THAs were functioning at the latest follow-up at 35 years or at the time of death [26]. The study performed by the Nordic Arthroplasty Register Association database reported 10-year survival of different cemented THAs implanted between 1995 and 2013 [27]. The most common types were Charnley (> 43,000), Lubinus (> 116,000), and Exeter (> 75,000). Several brands performed very well, e.g. Lubinus, Charnley, and Exeter yielding 95.7%, 94.1%, and 93.5% survival, respectively, at 10 years, but several other brands showed inferior performance. Good clinical outcomes of Charnley THAs were published by other short-term [28, 29] and long-term studies [30–33].

Clinical studies regarding cemented Charnley–Muller THAs are more scarce. Pavlov reported a follow-up of 512 consecutive THAs implanted between 1967 and 1972, with overall survival of 64% after 15 years and 43% after 17 years [4]. The main reason for the revision was aseptic loosening; the incidence of acetabular loosening increased with time, and that of femoral decreased. Femoral loosening was identified to be affected by several factors, such as prosthesis type and position, patient weight, activity level, and previous operation. Not all loose implants were, however, symptomatic [34, 35]. Radiographic loosening was commonly observed [36] with a 73% incidence of femoral looseness and a 22% incidence of progressive femoral loosening. “Looseness” was defined as a fracture of the acrylic cement and radiolucent lines of any size at the cement/stem and/or cement /bone interface. It was found in 121 out of 166 THAs [36]. “Loosening” was classified into four failure modes (from pistoning to weak proximal medial support). Thirty-one of 115 Charnley–Muller THAs examined at a mean of 5.9 years showed radiological evidence of femoral component loosening, and in 14 hips, loosening was evidenced [37]. In another study, 467 Charnley–Muller type THAs were operated between 1969 and 1974; 167 were reviewed with more than a 10-year follow-up [38]. Eighty-one per cent were satisfactory after the initial procedure.

## Conclusions

The revision percentage obtained in this study of 15.9% at 45 years represents excellent long-term survival of Alivium prostheses. Although the absence of the second endpoint, i.e. patient death, represents the limitation of this study to calculate the survivor curve, our study presents valuable data not reported previously for Charnley–Muller prostheses at 45 years of follow-up and a unique insight into the collected retrievals from the materials point of view. Polyethylene particles are identified as the main wear product released in periprosthetic tissue; the majority of particles (97%) were sized ≤ 0.3 µm. The shape of polyethylene particles isolated from the tissue corresponded to the wear damage of the retrieved polyethylene acetabular cups. Zirconia particles originating from the cement mantle were also isolated, whereas metal particles could be only rarely found, probably due to their small size. Histological features reflected a mild response to wear particles. This study confirms that the combination of the hospital arthroplasty register and retrieval analysis is a good basis for long-term surveillance of the THA’s performance, even for implant types that date several decades back.

## Data Availability

Data will be available on request.

## References

[1] Jackson J (2011). Father of the modern hip replacement: Professor Sir John Charnley (1911–82). J Med Biogr.

[2] Shah N, Porter M (2005). Evolution of cemented stems. Orthopedics.

[3] Callaghan JJ, Bracha P, Liu SS, Piyaworakhun S, Goetz DD, Johnston RC (2009). Survivorship of a Charnley total hip arthroplasty. A concise follow-up, at a minimum of thirty-five years, of previous reports. J Bone Joint Surg.

[4] Pavlov PW (1987). A 15-year follow-up study of 512 consecutive Charnley-Muller total hip replacements. J Arthroplasty.

[5] Campbell P, Ma S, Yeom B, McKellop H, Schmalzried TP, Amstutz HC (1995). Isolation of predominantly submicron-sized UHMWPE wear particles from periprosthetic tissue. J Biomed Mater Res.

[6] Milošev I, Trebše R, Kovač S, Cör A, Pišot V (2006). Survivorship and retrieval analysis of Sikomet metal-on-metal total hip replacements at a mean of seven years. J Bone Joint Surg Am.

[7] Milošev I (2017). From in vitro to retrieval studies of orthopedic implants. Corrosion.

[8] Hardinge K (1982). The direct lateral approach to the hip. J Bone Joint Surg (Br).

[9] McFarland B, Osborne G (1954). Approach to the hip: a suggested improvement on Koher′s method. J Bone Joint Surg (Br).

[10] Kaplan CJ (1961). Posterior approach to the hip joint in prosthetic replacement. S Afr Med J.

[11] https://www.ob-valdoltra.si/sl/international. Accessed 14 Mar 2023

[12] Catelas I, Bobyn D, Medley JB, Krygier JJ, Zukor DJ, Petit A, Huk OL (2001). Effects of digestion protocols on the isolation and characterization of metal-metal wear particles. I. Analysis of particle size and shape. J Biomed Mater Res.

[13] Doorn PF, Mirra JM, Campbell PA, Amstutz HC (1996). Tissue reaction to metal on metal total hip prostheses. Clin Orthop Relat Res.

[14] Ellison P, Hallan G, Johan P, Gjerdet NR, Havelin LI (2012). Coordinating retrieval and register studies improves postmarket surveillance. Clin Orthop Relat Res.

[15] Milošev I, Kovač S, Trebše R, Levašič V, Pišot V (2012). Comparison of ten-year survivorship of hip prostheses with use of conventional polyethylene, metal-on-metal, or ceramic-on-ceramic bearings. J Bone Joint Surg Am.

[16] Topolovec M, Cör A, Milošev I (2014). Metal-on-metal vs. metal-on-polyethylene total hip arthroplasty tribological evaluation of retrieved components and periprosthetic tissue. J Mech Behav Biomed Mater.

[17] McKellop H, Park SH, Chiesa R, Doorn P, Lu B, Normand P, Grigoris P, Amstutz H (1996). In vivo wear of three types of metal on metal hip prostheses during two decades of use. Clin Orthop Rel Res.

[18] Schmalzried TP, Huk OL (2004). Patient factors and wear in total hip arthroplasty. Clin Orthop Rel Res.

[19] Bracco P, Bellare A, Bistolfi A, Affatato S (2017). Ultra-high molecular weight polyethylene: Influence of the chemical, physical and mechanical properties on the wear behavior. A review Materials.

[20] Milošev I, Trebše R, Kovač S, Aoi T, Toshida A (2009). Materials development and latest results of various bearings for total hip arthroplasty. Hip replacement. Approaches, complications and effectiveness.

[21] Billi F, Benya P, Kavanaugh A, Adams J, Ebramzadeh E, McKellop H (2012). An accurate and sensitive method to separate, display, and characterize wear debris. Clin Orthop Rel Res.

[22] Milošev I, Strehblow H-H (2003). The composition of the surface passive film formed on CoCrMo alloy in simulated physiological solution. Electrochim Acta.

[23] Clauss M, Ilchmann T, Zimmermann P, Ochsner PE (2010). The histology around the cemented Müller straight stem. J Bone Joint Surg Br.

[24] Mullins MM, Norbury W, Dowell JK, Heywood-Waddington M (2007). Thirty-year results of a prospective study of Charnley total hip arthroplasty by the posterior approach. J Arthroplasty.

[25] Buckwalter AE, Callaghan JJ, Liu SS, Pedersen DR, Goetz DD, Sullivan PM, Leinen JA, Johnston RC (2006). Results of Charnley total hip arthroplasty with use of improved femoral cementing technique. J Bone Joint Surg Am.

[26] Warth LC, Callaghan JJ, Liu SS, Klaassen AL, Goetz DD, Johnston RC (2014). Thirty-five-year results after Charnley total hip arthroplasty in patients less than fifty years old. J Bone Joint Surg Am.

[27] Junnila M, Laaksonen I, Eskelinen A, Pulkkinen P, Havelin LI, Furnes O, Fenstad AM, Pedersen AB, Overgaard S, Kärrholm J, Garellick G, Malchau H, Mäkelä KT (2016). Implant survival of the most common cemented total hip devices from the Nordic Arthroplasty Register Association database. Acta Orthop.

[28] Garelick G, Malchau H, Herberts P (1999). The Charnley versus the Spectron hip prosthesis. Clinical evaluation of a randomized, prospective study of 2 different hip implants. J Arthroplasty.

[29] Ritter MA, Campbell ED (1987). Long-term comparison of the Charnley, Muller, and trapezoidal-28 total hip prostheses. J Arthroplasty.

[30] (PDF) Annual report 2021 Norwegian National Advisory Unit on Arthroplasty and Hip Fractures Norwegian Arthroplasty Register Norwegian Cruciate Ligament Register Norwegian Hip Fracture Register Norwegian Paediatric Hip Register (researchgate.net)

[31] Wroblewski BM, Siney BA (1993). Charnley low-friction arthroplasty of the hip. Long-term results Clin Orthop Rel Res.

[32] Older J (2002). Charnley low-friction arthroplasty. A worldwide retrospective review at 15 to 20 years. J Arthroplasty.

[33] Callaghan JJ, Templeton JE, Liu SS, Pederson DR, Goetz DD, Sullivan PM, Johnston RC (2004). Results of Charnley total hip arthroplasty at a minimum of thirty years. A concise follow-up of a previous report. J Bone Joint Surg Am.

[34] McBeath AA, Foltz RN (1979). Femoral component loosening after total hip arthroplasty. Clin Orthop Rel Res.

[35] McBeath AA, Schopler SA, Narechania RG (1980). Circumferential and axial strain in the proximal femur. Clin Orthop Rel Res.

[36] Cotterill P, Hunter GA, Tile M (1982). A radiographic analysis of 166 Charnley-Müller total hip arthroplasties. Clin Orthop Rel Res.

[37] Olsson SS, Jernberger A, Tryggö D (1981). Clinical and radiological long-term results after Charnley-Müller total hip replacement. Acta Orthop Scand.

[38] Kempf IJ, Henky P, Disteldorf M, Babin SR, Schvingt E (1986). 10-year follow-up of a homogeneous series of 467 total hip prostheses of the Charnley-Müller type. Study of 167 cases. Rev Chir Orthop Reparatrice Appar Mot.

